# Social vulnerability and cognitive functioning among older adults in the United States National Health and Nutrition Examination Survey

**DOI:** 10.1002/alz.089835

**Published:** 2025-01-09

**Authors:** Laura C Arboleda‐Merino, Erika Walker, Samuel D Fansler, Sung Kyun Park, Kelly M. Bakulski

**Affiliations:** ^1^ University of Michigan, Ann Arbor, MI USA; ^2^ Johns Hopkins, Baltimore, MD USA; ^3^ University of Michigan School of Public Health, Ann Arbor, MI USA

## Abstract

**Background:**

Numerous social factors, including socioeconomic status, education, and healthcare access, influence health outcomes in older adults. Despite the potential clinical utility of assessing social vulnerability, it is not routinely evaluated in clinical assessments of older adults. This study aims to investigate the association between cognitive decline and a comprehensive social vulnerability index, defined as an index comprising many social factors such as income, education, and housing tenure.

**Method:**

The study used data from the US National Health and Nutrition Examination Survey (NHANES) 1999‐2002 and 2011‐2014 cross‐sectional cycles. Cognition was assessed using the Digit Symbol Substitution Test (DSST, scores 0‐117) among participants 60+ years of age. Social vulnerability was assessed using a modified Social Vulnerability Index (SVI) based on components such as race/ethnicity, household income, education, health insurance, employment, housing tenure, and food security, with scores indicating the degree of social vulnerability ranging from 0 (least vulnerable) to 7 (most vulnerable). Associations of social vulnerability with cognitive function (adjusted for sex, age, and NHANES cycle) were analyzed by generalized linear models.

**Result:**

Participants with complete cognition and demographic measures (n = 5,272) were of mean age 70.5 years, 51.1% female, 68.0% had completed high school or above, and 55.0% were non‐Hispanic White. The mean DSST score was 43.3 (SD = 18.1), and 24.8% had low cognitive performance (lowest 25th percentile; DSST<31). The mean SVI was 2.55 (SD = 1.67). In adjusted analyses, each additional social deficit—a one unit increase in the Social Vulnerability Index—was associated with almost 6 points lower DSST score (β: ‐5.97; 95% Confidence interval (CI): ‐6.20, ‐5.74; p <0.0001).

**Conclusion:**

Our results suggest that increasing social vulnerability, defined by using a social vulnerability index incorporating many social factors, is associated with lower cognitive functioning among older adults. The results suggest the need for further investigation into the relationship between social vulnerability and cognition, with a focus on exploring potential interventions to mitigate its impact.

